# Nanotechnology in Sustainable Agriculture: Recent Developments, Challenges, and Perspectives

**DOI:** 10.3389/fmicb.2017.01014

**Published:** 2017-06-20

**Authors:** Ram Prasad, Atanu Bhattacharyya, Quang D. Nguyen

**Affiliations:** ^1^Amity Institute of Microbial Technology, Amity UniversityNoida, India; ^2^Department of Entomology, University of Agricultural Sciences, Gandhi Krishi Vigyan KendraBengaluru, India; ^3^Research Centre of Bioengineering and Process Engineering, Faculty of Food Science, Szent István UniversityBudapest, Hungary

**Keywords:** sustainable agriculture, nanotechnology, nanofertilizer, nanopesticides, nanoencapsulation, nanoemulsions

## Abstract

Nanotechnology monitors a leading agricultural controlling process, especially by its miniature dimension. Additionally, many potential benefits such as enhancement of food quality and safety, reduction of agricultural inputs, enrichment of absorbing nanoscale nutrients from the soil, etc. allow the application of nanotechnology to be resonant encumbrance. Agriculture, food, and natural resources are a part of those challenges like sustainability, susceptibility, human health, and healthy life. The ambition of nanomaterials in agriculture is to reduce the amount of spread chemicals, minimize nutrient losses in fertilization and increased yield through pest and nutrient management. Nanotechnology has the prospective to improve the agriculture and food industry with novel nanotools for the controlling of rapid disease diagnostic, enhancing the capacity of plants to absorb nutrients among others. The significant interests of using nanotechnology in agriculture includes specific applications like nanofertilizers and nanopesticides to trail products and nutrients levels to increase the productivity without decontamination of soils, waters, and protection against several insect pest and microbial diseases. Nanotechnology may act as sensors for monitoring soil quality of agricultural field and thus it maintain the health of agricultural plants. This review covers the current challenges of sustainability, food security and climate change that are exploring by the researchers in the area of nanotechnology in the improvement of agriculture.

## Introduction

Agriculture is always most important and stable sector because it produces and provides raw materials for food and feed industries. The limit of natural resources (production land, water, soil, etc.) and the growth of population in the world claim the agricultural development to be economically further, viable, environmentally and efficiently. This alteration will be the vital for achieving many factors in the recent year (Johnston and Mellor, 1961; Yunlong and Smit, 1994; Mukhopadhyay, 2014). Agricultural nutrient balances are differed noticeably with economic growth, and especially from this surmise, the development of the soil fertility is very much significant in developing countries (Campbell et al., 2014).

The development of agriculture is compulsory phenomena for the purge of poverty and hunger which must be getting rid of from the present situation. Therefore, we should have to take one bold step for agriculture development. In this world mainstream of peoples are below poverty level which are being scatted in the rural area where agriculture enlargement has not so being effective.

Nowadays, the most vital obsession is to create flanked by, agriculture poverty and nutritional process getting food. Therefore, new technology should have to adopt that decidedly focuses on getting better agricultural production (Yunlong and Smit, 1994). Recently, food and nutritional security are fully embedded in the novel knowledge. The agriculture development also depends on the social inclusion, health, climate changes, energy, ecosystem processes, natural resources, good supremacy, etc., must also be documented in specific target oriented goals. Therefore, sustainable agricultural strengthening the practical opportunity to get rid of poverty and hunger of the people. The agriculture on the road to recovery, thus the environmental performance is required and at the same time participation of food chain ecosystems are required in relation to agricultural food production (Thornhill et al., 2016).

**FIGURE 1:**
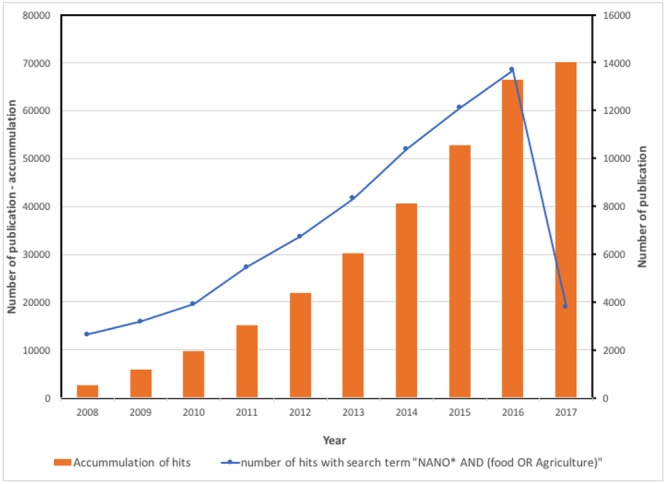
Number of documents on scopus.com with the search term “Nano and (food or agriculture). The hits were grouped annually. Accessed date: March 15, 2017.

**FIGURE 2:**
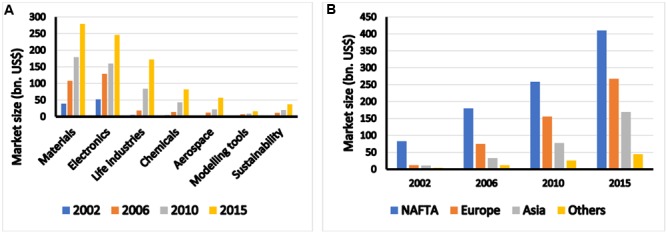
World market of nanotechnology by applications **(A)** and regions **(B)**. Source of data: Helmut Kaiser Consultancy (hkc22.com), Accessed date: March 15, 2017.

No doubt that the sustainable growth of agriculture totally depends on the new and innovative techniques like nanotechnology. Naturally, it haunts us to know what is this important technology? If we like to go in the year 1959 Feynman’s lecture on “Plenty of room at the bottom,” from this very day, the nanoprocess is in underway (Feynman, 1996). Later on Professor Norio Tanaguchi (1974) proposed the actual term of nanotechnology (Bulovic et al., 2004). Afterward, nanotechnology develops more vivid way, as because, more recent instruments develops to consider or isolate nanomaterials in accurate way (Bonnell and Huey, 2001; Gibney, 2015). Additionally, the number of publications related to the term of “nano” was also grown exponentially. **Figure 1** demonstrates the number of documents on scopus.com (accessed date: March 15, 2017) with the search term of “Nano and (Food and Agriculture).” In 2016, about 14,000 documents with nanotechnology in food or agriculture were listed meaning high activities of this field. Also about 2707 patents matched this criteria are found in world patent database^1^. The world market size of nanotechnology in 2002 was about US$ 110.6 billion and predicted to grow to US$ 891.1 billion in 2015 according to analysis of Helmut Kaiser Consultancy^2^. The developments of nanotechnology in materials and electronics have higher dynamics than other applications (**Figure 2A**). Recently, food and agriculture also require high amount of nanomaterials especially in packaging. The NAFTA region shares the biggest slice from the market size (**Figure 2B**), but Europe and Asia especially China, Japan, and India also come up very dynamically.

It is well known that one billionth of a meter is one nanometer (nm). Why this nano will change its property? It is due to alteration in the atoms and develops a magnetic power. It can be projected that the smaller size of nanomaterial possesses larger surface area and exhibits more active. With this course of action nanotechnology is knocking the doors of perception. The magnetic property of polymer develops due to tellurium atoms; antimony-bismuth; and sulfur atoms. Moreover, it has been observed that when the atoms of do pant and atoms of europium interact together, and then the entire molecules carry out the magnetic property. Thus the alter property of nanomaterials is related with more reactive in the most sectors including in biological process (Pokropivny et al., 2007; Prasad, 2014). This ultimate technology possesses several unique electronic association, plasmonic and optical properties which are related with the quantum confinement effects, the alteration of the electronic energy levels may appear due to the surface area in relation to volume ratio (Sun, 2007; Aziz et al., 2015; Prasad et al., 2016). In the present century, there is a big demand for fast, reliable, and low-cost systems for the detection, monitoring, and diagnosis for biological host molecules in agricultural sectors (Vidotti et al., 2011; Sagadevan and Periasamy, 2014). The application of chemically synthesize nanomaterials now a days considered as toxic in the nature, in attract of this, nanomaterials may synthesis from plant system and it considered as green nanotechnology (Prasad, 2014). Green nanotechnology is a safe process, energy efficient, reduces waste and lessens greenhouse gas emissions. Use of renewable materials in production of such products is beneficial, thus these processes have low influence on the environment (Prasad et al., 2014, 2016). Nanomaterials are eco-environmentally sustainable and significant advances have been made in the field of green nanotechnology. In the present decade, it is more shift toward the green nano in a faster rate for implementation its functions. Still it is not clear how the environmental sustainability of green nanotechnology will be achieved in future? These risks must be mitigated in advancing green nanotechnology solutions (Kandasamy and Prema, 2015).

In modern agriculture, sustainable production and efficiency are unimaginable without the use of agrochemicals such as pesticides, fertilizers, etc. However, every agrochemical has some potential issues including contamination of water or residues on food products that threat the human being and environmental health, thus the precise management and control of inputs could allow to reduce these risks (Kah, 2015). The development of the high-tech agricultural system with use of engineered smart nanotools could be excellent strategy to make a revolution in agricultural practices, and thus reduce and/or eliminate the influence of modern agriculture on the environment as well as to enhance both the quality and quantity of yields (Sekhon, 2014; Liu and Lal, 2015).

The development of biosensors is also a good field for exploitation of many strengths of nanotechnology, thus nanotechnology is there and plays an essential role. Due to special properties of nanomaterials, on one hand, the sensitivity and performance of biosensors could be improved significantly in their applications (Fraceto et al., 2016); on another hand, many new signal transduction technologies are let to be introduced in biosensors (Sertova, 2015). Additionally, use of nanomaterials let to miniaturize many (bio)sensors to small and compact/smart devices such as nanosensors and other nanosystems that are very important in biochemical analysis (Viswanathan and Radecki, 2008; Sertova, 2015; Fraceto et al., 2016). It also helps to detect the mycotoxins present in several foods and their functions are very rapid (Sertova, 2015).

## Nanoparticles and Their Functions

### Carbon Nanotubes (CNTs)

It is a new form of carbon, equivalent to two dimensional graphene sheet rolled into a tube. Two main types of nanotubes are single-walled nanotubes (SWNTs) and multi-walled nanotubes (MWNTs). Its tensile strength ∼200 GPa, thus ideal for reinforced composites and nanoelectro mechanical systems. Moreover, metallic or semiconducting and offers amazing possibilities to create electronic circuits, or even complete nanodevices. Structurally, the nanotube systems consist of graphitic layers seamlessly wrapped into cylinders. Recently, fluorescent nanoparticles (NPs) or quantum dots (QDs) have been developed for labeling the plant proteins (Pyrzynska, 2011; Chahine et al., 2014).

No doubt that properties (mechanical, electronic, thermal, optical, elastic, etc.), and thus applicability of CNTs were determined by geometrical dimensions especially by diameter. Diameter of most SWNTs is about 1 nm and strongly correlated to synthesis techniques, mixing of σ and α bonds and electron orbital rehybridization. Exploitation of these properties of CNTs definitely will open new possibilities to develop many types of nanodevices which confers unique conductive, optical and thermal properties for applications in agri-field and in development of sustainable agricultural conditions (Raliya et al., 2013). Agrochemicals or other substances can be targeted to hosts by delivery systems based on CNTs, thus led to reduce the amount of chemicals released into the environment as well as the damage to other plant tissues (Raliya et al., 2013; Hajirostamlo et al., 2015).

Camilli et al. (2014) reported that the absorption of the toxic organic solvent dichlorobenzene from water increased about 3.5 times by some CNTs nano-sponges than CNT powder. Generally, the use of CNT nano-sponges containing sulfur and iron increases efficiency in soaking up water contaminants such as pesticides, fertilizers, oil, and pharmaceuticals. Unfortunately, under certain circumstances CNTs may cause vitality of human cells by penetrability and accumulability in the cytoplasm (Porter et al., 2007).

### Quantum Dots

Generally, semiconductor QDs are high quantum yield and molar extinction coefficients, broad absorption spectra with narrow, symmetric fluorescence spectra spanning the ultraviolet to near-infrared, large effective excitation, high resistance to photobleaching and exceptional resistance to photochemical degradation. Thus these are excellent fluorescence, quantum confinement of charge carrier’s materials and possess size tunable band energy (Bulovic et al., 2004; Androvitsaneas et al., 2016). QDs have unique spectral properties compared with traditional organic dyes, thus recently, they have been applied as a new generation of fluorophores in bioimaging and biosensing (Bakalova et al., 2004). QDs also function as photocatalysts for the light driven chemical conversion of water into hydrogen as a pathway to solar fuel (Konstantatos and Sargent, 2009). QDs at low concentration revealed no detectable cytotoxicity for seed germination and seedling growth. Therefore, based on this transport approach, QDs can be utilized for live imaging in plant root systems to verify known physiological processes (Hu et al., 2010; Das et al., 2015).

### Nanorods

Multifunctional plasmonic materials which can couple sensing phenomenon well and size tunable energy regulation, can be coupled with MEMS, and induce specific field responses (Bulovic et al., 2004). The gold nanorods significantly physiological changes occurred of watermelon plant and confirmed phytotoxicity toward plant particularly at high concentration (Wan et al., 2014) and also ability to transport auxin growth regulator 2,4-D, which resulted in a significant influence on the regulation of tobacco cell culture growth (Nima et al., 2014).

### Micro- and Nanoencapsulation

Encapsulation is defined as process in which the given object is surrounded by a coating or embedded in homogeneous or heterogeneous matrix, thus this process result capsules with many useful properties (Rodríguez et al., 2016). The benefits of encapsulation methods are for protection of substances/objects from adverse environments, for controlled release, and for precision targeting (Ezhilarasi et al., 2012; Ozdemir and Kemerli, 2016).

Depending on size and shape of capsules different encapsulation technologies are mentioned, while the (macro) encapsulation/coating results capsules in macroscale, whereas the micro- and nanoencapsulation will give particles in micro- and nanoscale size (Ozdemir and Kemerli, 2016). Nanocapsules are vesicular systems in which the substances are confined to a cavity consisting of an inner liquid core enclosed by a polymeric membrane (Couvreur et al., 1995). Recently, micro and NPs are getting significant attention for delivery of drugs, for protection and increase in bioavailability of food components or nutraceuticals, for food fortification and for the self-healing of several materials, and also it possesses big prospective phenomenon in plant science (Ozdemir and Kemerli, 2016). Some drugs such as peptides or anti-inflammatory compounds are successfully nanoencapsulated (Puglisi et al., 1995; Hildebrand and Tack, 2000; Haolong et al., 2011). The development of nanoencapsulated methods for ligation of targeted tissues to NPs which will make possible to deliver several biologically active compounds to the target tissues (Pohlmann et al., 2008). Furthermore, the development of this technology will build more possibility to create new drugs with precise therapeutic action on embattled tissues. Nanocapsules can potentially be used as MRI-guided nanorobots or nanobots (Vartholomeos et al., 2011).

### Nanoemulsions

Nanoemulsions are formed by very small emulsion nanoscale droplets (oil/water system) exhibiting sizes lower than ∼100 nm (Gutiérrez et al., 2008; Anton and Vandamme, 2011). Although fundamentally significant differences between nanoemulsions and microemulsions could not be exists, but in fact, the physical properties of nanoemulsions can be quite different from those of microscale emulsions (Mason et al., 2006; Gupta et al., 2016). Due to the size of droplets, the ratio of surface area to volume, Laplace pressure and elastic modulus of nanoemulsions are significantly larger than that of ordinary emulsions. Moreover unlike general emulsions, most of nanoemulsions appear optically transparent that, thus, technically have many advantages make such us incorporation into drinks. Unfortunately, the formulation of nanoemulsion needs very high energy, thus it requires some special devices that are able to generate extreme shear stress such as, high pressure homogenizator or ultrasonic generator (Asua, 2002; Gupta et al., 2016). Tadros et al. (2004) reported “low-energy” method for formation of nanoemulsions and in this process, two liquid phases (one is a homogeneous liquid consisted of lipophilic phase and hydrophilic surfactant plus potentially a solvent, polymer or drug, and the other is an aqueous phase, even pure water) are bought into contact of this phase. Then the hydrophilic species contained in the oily phase is rapidly solubilized into the aqueous one, inducing the demixation of the oil in the form of nano-droplets, instantly stabilized by the amphiphiles (Anton and Vandamme, 2009; Gupta et al., 2016). This method thus is seemed to be simplest and does not require any special devices with high energy.

## Nanotechnology and Agricultural Sustainable Development

The nanotechnology can takes an important part in the productivity through control of nutrients (Gruère, 2012; Mukhopadhyay, 2014) as well as it can also participate in the monitoring of water quality and pesticides for sustainable development of agriculture (Prasad et al., 2014). Nanomaterials have such diverse assets and activities that it is impossible to deliver a general assessment of their health and environmental risks (Prasad et al., 2014). Properties (other than size) of NPs have the influence on toxicity include chemical composition, shape, surface structure, surface charge, behavior, extent of particle aggregation (clumping) or disaggregation, etc. may associate with engineered NPs (Ion et al., 2010). For this reason even nanomaterials of the same chemical composition that have different sizes or shapes can exhibit their different toxicity. The implication of the nanotechnology research in the agricultural sector is become to be necessary even key factor for the sustainable developments. In the agri-food areas pertinent applications of nanotubes, fullerenes, biosensors, controlled delivery systems, nanofiltration, etc. were observed (Ion et al., 2010; Sabir et al., 2014). This technology was proved to be as good in resources management of agricultural field, drug delivery mechanisms in plants and helps to maintain the soils fertility. Moreover, it is being also evaluated steadily in the use of biomass and agricultural waste as well as in food processing and food packaging system as well as risk assessment (Floros et al., 2010). Recently, nanosensors are widely applied in the agriculture due to their strengths and fast for environmental monitoring of contamination in the soils and in the water (Ion et al., 2010). Several sensors based on nano-detection technology such as viz. biosensors, electrochemical sensors, optical sensors, and devices will be the main instruments for detecting the heavy metals in trace range (Ion et al., 2010).

Nanomaterials not only directly catalyze degradation of waste and toxic materials but it also aids improve the efficiency of microorganisms in degradation of waste and toxic materials. Bioremediation uses living organisms to break down or remove toxins and harmful substances from agricultural soil and water. In particular, some other terms are also generally used such as bioremediation (beneficial microbes), phytoremediation (plants), and mycoremediation (fungi and mushrooms). Thus, with the bioremediation the heavy metals can be removed from soil and water environmentally and efficiently by microorganisms (Dixit et al., 2015). Therefore, the agricultural bioremediation helps in sustainable remediation technologies to resolve and restore the natural situation of the soil. It is an interesting phenomena in considering the nano–nano interaction to remove the toxic component of the agricultural soil and make it sustainable (Ion et al., 2010; Dixit et al., 2015).

### Nanofertilizers

In the recent decade nanofertilizers are freely available in the market, but particularly the agricultural fertilizers are still not shaped by the major chemical companies (**Table 1**). Nanofertilizers may contain nano zinc, silica, iron and titanium dioxide, ZnCdSe/ZnS core shell QDs, InP/ZnS core shell QDs, Mn/ZnSe QDs, gold nanorods, core shell QDs, etc. as well as should endorse control release and improve the its quality. Studies of the uptake, biological fate and toxicity of several metal oxide NPs, *viz.* Al_2_O_3_, TiO_2_, CeO_2_, FeO, and ZnONPs were carried out intensively in the present decade for agricultural production (Dimkpa, 2014; Zhang et al., 2016). The deficiency of zinc has been documented as one of the main problems in limiting agricultural productivity in the alkaline nature of soils (Sadeghzadeh, 2013).

**Table 1:** Some commercial product of nanofertilizers.

Commercial product	Content	Company
Nano-Gro^TM^	Plant growth regulator and immunity enhancer	Agro Nanotechnology Corp., FL, United States

Nano Green	Extracts of corn, grain, soybeans, potatoes, coconut, and palm	Nano Green Sciences, Inc., India

Nano-Ag Answer^^®^^	Microorganism, sea kelp, and mineral electrolyte	Urth Agriculture, CA, United States

Biozar Nano-Fertilizer	Combination of organic materials, micronutrients, and macromolecules	Fanavar Nano-Pazhoohesh Markazi Company, Iran

Nano Max NPK Fertilizer	Multiple organic acids chelated with major nutrients, amino acids, organic carbon, organic micro nutrients/trace elements, vitamins, and probiotic	JU Agri Sciences Pvt. Ltd, Janakpuri, New Delhi, India

Master Nano Chitosan Organic Fertilizer	Water soluble liquid chitosan, organic acid and salicylic acids, phenolic compounds	Pannaraj Intertrade, Thailand

TAG NANO (NPK, PhoS, Zinc, Cal, etc.) fertilizers	Proteino-lacto-gluconate chelated with micronutrients, vitamins, probiotics, seaweed extracts, humic acid	Tropical Agrosystem India (P) Ltd, India

Metal oxide NPs are radiolabeled by direct proton bombardment or enriched during synthesis with ^18^O to generate ^18^F (Llop et al., 2014). Size, degree of aggregation and zeta potential of the metal oxide NPs are studied in the presence of proteins and cell media (Llop et al., 2014; Marzbani et al., 2015). Moreover, NP uptake and intracellular fate are followed by ion beam microscopy, transmission electron microscopy, Raman chemical imaging spectroscopy, and confocal laser scanning microscopy (Marzbani et al., 2015). In the future, sustainable bio-based economy that uses eco-efficient bio-processes and renewable bio-resource, will continue decrease and substitute the harmful materials in established applications, and thus it will play a major role (the key strategic challenge) in the development of the technologies desired to address to 21st century (Prasad et al., 2014; Marzbani et al., 2015). Accumulation of knowledge in fields of ecology, biology, biodiversity, material science, biotechnology, and engineering opens possibilities to increase biomass productivity as well as to utilize biomass and organic wastes at a highly efficient.

In the present century, the smart agriculture is a way to achieve priority of short and long term development in the countenance of climate change and serves as a link to others (Helar and Chavan, 2015). It seeks to support countries and other functional aspects in securing the necessary agricultural functions (Kandasamy and Prema, 2015). In the past few years, researches related to the expansion of resources in a nanometric extent and their inherent properties are intensively conducted and focused. Practically, when the crystallite size of inorganic materials are reduced to nanoscale, two different phenomena can occur. In the first one (quantum size effect), radical changes of the physical–chemical properties of material are observed. In this case, the performance is totally dependent on the semiconductors-NPs. On the other hand, due to the huge ratio of surface area to volume, NPs exhibit very good transduction properties which are being more interesting for analytical purpose of agricultural products (Kandasamy and Prema, 2015). Nanostructures materials exposed several advantages in logical sciences when used as transducers or as a part of the appreciation in a macro-sized sensing device. In this facts the gold NPs (AuNPs) has its intrinsic properties, and may use as transducers for several improvements of agricultural products. The AuNPs have well-known surface plasmon band that is visible around 520 nm. Moreover, AuNPs have high surface areas and distinctive physicochemical assets that can be easily tuned and thus making them ideal candidates for developing biosensing devices. Additionally, these NPs possess attracted attention in biological studies owing to their low toxicity, biocompatibility and unique optical properties. Biological tests measuring the presence or activity of selected analytics become quicker, more sensitive and flexible when nanoscale particles are put together (Vidotti et al., 2011; Kandasamy and Prema, 2015). Thus, application of nanoscale particles results numerous advantages over traditional procedures.

### Nanopesticides

The use of nanomaterials in plant protection and production of food is under-explored area in the future. It is well known that insect pests are the predominant ones in the agricultural fields and also in its products, thus NPs may have key role in the control of insect pests and host pathogens (Khota et al., 2012; **Table 2**). The recent development of a nanoencapsulated pesticide formulation has slow releasing properties with enhanced solubility, specificity, permeability and stability (Bhattacharyya et al., 2016). These assets are mainly achieved through either protecting the encapsulated active ingredients from premature degradation or increasing their pest control efficacy for a longer period. Formulation of nanoencapsulated pesticides led to reduce the dosage of pesticides and human beings exposure to them which is environmentally friendly for crop protection (Nuruzzaman et al., 2016). So, development of non-toxic and promising pesticide delivery systems for increasing global food production while reducing the negative environmental impacts to ecosystem (de Oliveira et al., 2014; Kah and Hofmann, 2014; Bhattacharyya et al., 2016; Grillo et al., 2016).

**Table 2:** A list of studies on nanopesticides/herbicides and its application.

Carrier system	Agent	Purpose	Method	Reference
Chitosan	Imazapic and Imazapyr	Cytotoxicity assays	Encapsulation	Maruyama et al., 2016

Silica	Piracetam, pentoxifylline, and pyridoxine	Perfused brain tissue	Suspension	Jampilek et al., 2015

Alginate	Imidacloprid	Cytotoxicity, sucking pest (leafhoppers)	Emulsion	Kumar et al., 2014

Polyacetic acid-polyethylene glycol-polyacetic acid	Imidacloprid	Decrease the lethal concentration	Encapsulation	Memarizadeh et al., 2014

Carboxymethyl chitosan	Methomyl	Control release for longer time-period	Encapsulation	Sun et al., 2014

Chitosan/tripolyphosphate	Paraquat	Lower cyto- and genotoxicity	Encapsulation	Grillo et al., 2014

Chitosan/tripolyphosphate Chitosan-saponin Chitosan-Cu	Chitosan, saponin, CuSO_4_	Antifungal activity	Cross-linking	Saharan et al., 2013

Xyloglucan/poloxamer	Tropicamide	Have significantly higher corneal permeation across excised goat cornea Less toxic and non-irritant	Encapsulation	Dilbaghi et al., 2013

Wheat gluten	Ethofumesate	Reduce its diffusivity	entrapment/extrusion	Chevillard et al., 2012

Alginate	Azadirachtin	Slower release	Encapsulation	Jerobin et al., 2012

Surfactants/oil/water	Glyphosate	Increase in bio-efficacy, alleviating the negative effect of pesticide formulations into environment	Emulsion	Jiang et al., 2012

Alginate/chitosan	Paraquat	Increased period of action of the chemical on precise targets, while reducing problems of ecological toxicity	Pre-gelation of alginate then complexation between alginate and chitosan	Silva Mdos et al., 2011

Polyhydroxybutyrate-co-hydroxyvalerate	Atrazine	Decreased genotoxicity and increased biodegradability	Encapsulation	Grillo et al., 2010

Organic-inorganic nanohybrid	2,4-Dichlorophenoxyacetate	Controlrelease	Self-assembly	Hussein et al., 2005

Microencapsulation-like nanoencapsulation is used to develop the quality of products of desired chemicals delivery to the target biological process. Recently, few chemical companies openly promote nanoscale pesticides for sale as “microencapsulated pesticides.” Some products from Syngenta (Switzerland) such as Karate ZEON, Subdue MAXX, Ospray’s Chyella, Penncap-M, and microencapsulated pesticides from BASF may fighting fit for nanoscale (Gouin, 2004). Syngenta also markets in the Australia some products such as the Primo MAXX, Banner MAXX, Subdue MAXX, etc. Despite they are known as microemulsions in the market, however, they are really nanoscale emulsions. It confirms very thin interface between the term of microemulsion and nanoemulsion. This technique is commonly used for formulations of organic NPs (Gouin, 2004) containing active agrochemicals or substances of interest.

### Ecotoxicological Implications of the Nanoparticles

The advancement of nanotechnologies has presented significant extents of manufactured NPs into the environment. In order to protect human health and plant from the prospective antagonistic effects of a wide range of nanomaterials, an increasing number of research have focused on the assessment of the toxicity of the NPs normally used in industry (Yang and Watts, 2005; Rana and Kalaichelvan, 2013; Du et al., 2017; Tripathi et al., 2017a,b,c). The toxicity of a metal depends upon several factors like solubility, binding specificity to a biological site, and so forth. Metal NPs exhibit antibacterial, anticandidal, and antifungal activities (Aziz et al., 2016; Patra and Baek, 2017). Metal NPs exert cytotoxicity depending on the charge at membrane surface, of course, the efficiency of nanotoxic effects of NPs are definitely depending on structure of targeted cell-wall, thus the sensitive order should be mould > yeast > Gram-negative > Gram-positive. Nanotoxicity may be accredited to electrostatic interaction between NPs with membrane and their accumulation in cytoplasm (Rana and Kalaichelvan, 2011; Aziz et al., 2015, 2016).

Several NPs (TiO_2_, ZnO, SiO_2_, and Fullerenes) are photochemically active. When they are exposed to light, the excited electrons are generated that then form superoxide radicals in the presence of oxygen by direct electron transfer (Hoffmann et al., 2007). Thus, this ecotoxicity is surprised in the act when organisms are simultaneously exposed to NPs and UV light (particularly UV light has higher energy than visible light). In this case, the cells respond to oxidative stress by increasing a number of protective enzymatic or genetic constitutions that can easily be measured (Kovochich et al., 2005; Vannini et al., 2014), thus generation of reactive oxygen species (ROS) is oxidative stress parameter that can be exploited in determination of the context of toxicity and ecotoxicity. *In vitro* studies on the toxicity of NPs have confirmed the generation of ROS, for example, by TiO_2_ and fullerenes (Sayes et al., 2004), while on other hand, some authors revealed that NPs (fullerenes and silicon NPs) may protect against oxidative stress (Daroczi et al., 2006; Tripathi et al., 2016b, 2017d; Venkatachalam et al., 2017). Much more researches related to interactions between cells and NPs as well as mechanistic facets of NPs metabolism in organisms and specific cells are needed to clarify this dichotomy.

Ecotoxicological research would increasingly attention on the environmental consequence of the materials and complexity of natural systems. Extensive research would be necessary to determine delayed impacts of environmental exposure to NPs and to help determine possible adaptive mechanisms (Cox et al., 2017; Singh et al., 2017). More research on bioaccumulation in the food chain and interaction of NPs with other pollutants in the environment. NPs in plants enter cellular system, translocate them shoot and accumulate in various aerial parts, the possibility of their cycling in the ecosystem increases through various trophic levels. After accumulation of NPs effect rate of transpiration, respiration, altering the process of photosynthesis, and interfere with translocation of food material (Shweta et al., 2016; Tripathi et al., 2016b; Du et al., 2017). The degree of toxicity is linked to this surface and to the surface properties of the NPs. The ecotoxicity of NPs is thus very important as it creates a direct link between the adverse effects of NPs and the organisms including microorganisms, plants, and other organisms including humans at various trophic levels (Rana and Kalaichelvan, 2013; Tripathi et al., 2016a).

### Growth of Cultivated Plants and its Ecotoxicological Sustainability

**FIGURE 3:**
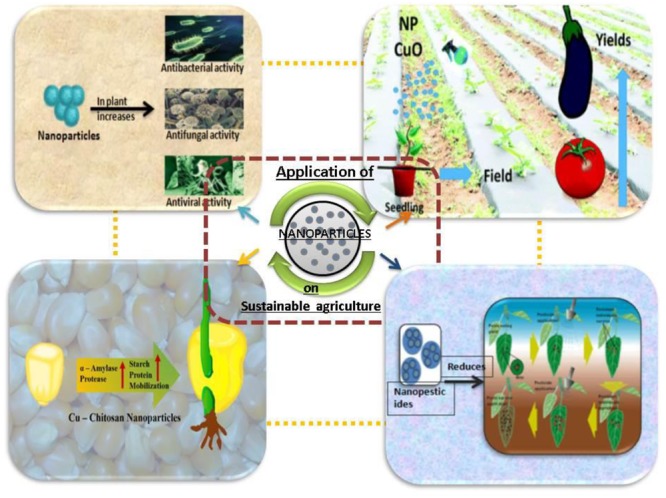
Potential applications of nanotechnology representing the consequences of nanoparticles in sustainable agriculture.

The agriculture host plants take the main part in food chain. Recently, the plants do not only grow on agricultural lands, but they are also developed on aqueous medium too. Naturally, several NPs of iron oxide (magnetite), a magnetic form of iron ore can deposit in the plant host. It is interesting to propose that the iron (II, III) oxide NPs (Fe_3_O_4_ -NPs) have the ability to accumulate in *Lepidium sativum* and *Pisum sativum* plants. Therefore, this type of observation clearly proposes that the roles to mention NPs are present in the natural ecosystem (Bystrzejewska-Piotrowska et al., 2012; Abbas et al., 2016). Moreover, the uses of polymeric NPs in the agricultural field, especially loaded with insecticides of plant origin are unique and increasingly permeated (Chakravarthy et al., 2012; Perlatti et al., 2013). No doubt that microorganism plays crucial role in maintaining soil health, ecosystem, and crop productivity (Mishra and Kumar, 2009). Therefore, it is very essential to know the ecotoxicological aspects of the considered agricultural field. If nanomaterials containing agricultural plants are devoid of any toxic nanocomposite, then the unique possibility of more production of agricultural crops. Thus, the introduction of engineered (either chemical or green) NPs in the agricultural field should always be a routine check-up to sustain an eco-friendly in the agricultural field (**Figure 3**).

## Nanobiosensors

Many advantages of physical–chemical properties of nanoscale materials are also exploitable in field of biosensors development of biosensors. Sagadevan and Periasamy (2014) stated that the sensitivity and performance of biosensors can be improved by using nanomaterials through new signal transduction technologies. The tremendous advancement in the nanobiosensors are due to the great technological demand for rapid, sensitive and cost-effective nanobiosensor systems in vital areas of human activity such as health care, agriculture, genome analysis, food and drink, the process industries, environmental monitoring, defense, and security. At present, the nanotechnology-based biosensors are at the early stage of development (Fogel and Limson, 2016). The improvement of tools and procedures used to fabricate, measure and image nanoscale objects, has led to the development of sensors. The nanomaterials such as metal (gold, silver, cobalt, etc.) NPs, CNT, magnetic NPs, and QDs have been actively investigated for their applications in biosensors which have become a new interdisciplinary frontier between biological detection and material science. Thus a biosensor is a device that combines a biological recognition element with physical or chemical principles. It integrates a biological one with an electronic component to yield a measurable signal component, and the biological recognition is through the transducer process and the signal processing through electronic achievement. The higher specificity and sensitivity of biosensor systems over the conventional methods are due to the presence of the bioreceptor (biological element) that is combined with a suitable transducer which produces a signal after interaction with the target molecule of interest. Recently, different natural and artificial bioreceptors are developed and applied such as enzymes, dendrimers, thin films, etc. Therefore, by biosensor, an analytical device, this converts a biological response into an electrical signal. It is concerned with these parts of biological elements like, an antibody, an enzyme, a protein, or a nucleic acid. The transducer and the associated electronics or signal processors that are primarily responsible for detection of the functions (Rai et al., 2012). The micro cantilever-based DNA biosensor that uses AuNPs have been developed and used widely to detect low level DNA concentration during a hybridization reaction (Brolo, 2012).

## Nanotechnologies in Food Industry

Nanoscale biosensors can take part in pathogen detection and diagnosis. Nanotechnology has the ability to supply bioactive ingredients in foodstuffs to hosts while improvement of knowledge of food materials at the nanoscale (Martirosyan and Schneider, 2014). It also helps in nanoscale filtration systems for improved texture modification of food. Nano biosensors interact with food, attractive surface and thus maintain the glaziers and colors of food, magnetic nanocomposite for tag sensors. Nanoprinted, intelligent packaging, controlled release (Ghaani et al., 2016), nano-additives (Khond and Kriplani, 2016), nanocoding of plastics and paper materials (Bhushani and Anandharamakrishnan, 2014), for authentication and identification purposes (Khond and Kriplani, 2016; Savina et al., 2016). In food quality tests, some important aspects should be covered such as sensing ability of label and package, *in situ* sensors, food quality monitoring (e.g., color, smell, taste, texture), control and nutraceuticals delivery, portable DNA/protein chips, etc. Most of the time, nanomaterials produced by bottom-up methods (Siegrist et al., 2008).

### Food Process

Recently nanotechnology is widely applied in food processing such as nanocarrier systems for delivery of nutrients and supplements, organic nano-sized additives for food, supplements, and animal feed. Many food products already contain NP naturally. Milk contains casein, a form of milk protein present at the nanoscale or meat is made up of protein filaments that are also be classified into nanomaterial group. The texture and properties of these products are determined by the organization and structures of proteins inside.

Recently, some nutrients mainly vitamins are encapsulated and delivered into the bloodstream through digestion system with very high efficiency. Some foods and drinks were fortified with these NPs without affecting the taste or appearance. NP emulsions are being used in ice cream and spreads of this nanoemulsion can improve the texture and uniformity of the ice cream (Berekaa, 2015). A real example: KD Pharma BEXBACH GMBH (Germany) provides encapsulated Omega-3 fatty acids in two different forms—suspension and powder. The capsulation technology used the resulted particles in nano- and microscale.

### Food Packaging and Labeling

In the food industry, maintaining some important factors such as quality, safe, freshness, taste, etc. whole supply chain requires producers to packaging and labeling their products. Development of smart packages that can provide useful information is still big challenge for researchers and producers. Recently, some packaging materials incorporated with “nanosensors” to detect the oxidation process in food have been produced and used in food industry. Working scheme is quite simple: when the oxidation occurs in the food package, NP-based sensors indicate the color change and information about the nature of the packed foods can be observed. This technology have been successfully applied in package of milk and meat (Bumbudsanpharoke and Ko, 2015).

Due to nanostructure, NPs are good barriers for diffusion gases such as oxygen, carbon dioxide, thus it can be exploited in food packaging. Some drinks (beer, soda waters, etc.) naturally have to keep appreciate amount of carbon dioxide can be packaged in the bottles made with nanocomposites because of minimization of CO_2_ lost, decrease in weight of packaging materials, increase in shelf life, etc. Other exploitation way is the incorporation of NPs in packaging and this technology will slow down some biochemical processes such as oxidation, degradation, etc. thus it help to extend the shelf-life of food products. In the food packaging industry, the most used materials are plastic polymers that can be incorporated or coated with nanomaterials for improved mechanical or functional properties (Berekaa, 2015). Moreover, nanocoatings on food contact surfaces act as barrier or antimicrobial properties. Silver NPs have been successfully embedded in the plastic for making food storage bins, and this acts like disinfection of bins, thus minimizing harmful bacterial growth. Therefore, the nanotechnology is a forward looking process, it acts as an agricultural biosecurity (Bumbudsanpharoke and Ko, 2015).

NPs in precise have revealed broad-spectrum antibacterial properties against both Gram-positive and Gram-negative bacteria. ZnO NPs were found to inhibit *Staphylococcus aureus* (Liu et al., 2009) and AgNPs exhibit concentration-dependent antimicrobial activity against *Escherichia coli, Aeromonas hydrophila*, and *Klebsiella pneumoniae* (Aziz et al., 2016). The antimicrobial mechanism of action of NPs is typically considered as of few prototypes such as oxidative stress and cell damage, metal ion release, or non-oxidative mechanisms (Wang et al., 2017). These mechanisms can happen concurrently. Firm studies have suggested that Ag NPs quick neutralization of the surface electric charge of the bacterial membrane and change its permeability, ultimately leading to apoptosis. Moreover, the generation of ROS prevents the antioxidant defense system and causes physiochemical damage to the intrinsic cell membrane. According to current investigation, the major processes causal the antibacterial effects of NPs are as follows: disruption of the bacterial cell membrane; generation of ROS; penetration of the bacterial cell membrane by passive or facilitated diffusion and induction of intracellular antibacterial effects, including interfaces with DNA replication, and inhibition of protein synthesis (Aziz et al., 2015; Wang et al., 2017).

Nanosensors help in food labeling and in combination with NP-based intelligent inks or reactive nanolayers may provide smart recognition of relevant food product. Printed labels in the food package that can indicate the following highlights: temperature, time, pathogens, freshness, humidity, etc. Nanobarcode particles with different patterns of gold which can form template and also with silver stripes are possible to synthesize itself. Lastly, we can suggest that the contaminant or nutrient sorption on NPs surfaces has attracted the attention of researchers for more studies on soil chemistry, showing that NPs have high sorption capacities for metal and anionic contaminants (Li et al., 2016). It was found that the contaminant sequestration was accomplished mainly by surface complication. It is likely that the sorbet surface species can be encapsulated within interior surfaces of NPs. A phenomenon with significant consequences for contaminant dispersion or remediation processes can exhibit. Moreover, metallic species as Ni can be linked to natural short-ordered aluminosilicates, TiO_2_ surfaces, humic acids, and aromatic compounds by MWCNTs and these association may be considered as very potent bioremediation in nano agricultural system (Raliya et al., 2013; Hajirostamlo et al., 2015).

## Future Perspectives

Sustainable agriculture must be taken as an ecosystem method, where abiotic–biotic-living beings live in accord with a co-ordinated stability of food chains and their related energy balances. New technologies, modernization, increased in use of nano-chemicals, specialization and government policies are adapted to maximize the production in agriculture. To overcome the situation, it is mandatory to establish the recent technology in the food industry. Therefore, the new and future technology is nanotechnology that possesses very unique property in food supply chain (from the field to table: crop production, use of agro-chemicals such as nanofertilizers, nanopesticides, nanoherbicides, etc., precision farming techniques, intelligent feed, enhancement of food texture and quality, and bioavailability/nutrient values, packaging and labeling, etc.) round the world agricultural sector. Some focused areas may need more attention in near future researches in the field of agricultural nanotechnology or nanofoods:

• New environmental and safety delivery systems for carrying special food/feed compounds, plant nutrients, etc. These systems also can have pharmaceutical application potentials.
• The (bio)sensors related nanotechnology have effective role in insect pest control and food products of agriculture. Consumers always can get actual information of the state of certain food product via intelligent food packaging corporated with nanosensors.
• The properties of nanomaterials such as size, dose, exposure time, surface chemistry, structures, immune response, accumulation, retention time, etc., and other effects should be accessed carefully. New analytical methods are needed to develop to detect, validate and access the effects of each nanomaterials/nanofoods in whole ecosystems. Life-cycle analysis of nanomaterials/nanofoods should be done. Improvement of wide-ranging databank as well as international collaboration for policy, idea and regulation are needed for manipulation of this knowledge. Additionally, the authorities should provide clear guidelines and roadmaps for reducing risks of the use of nanotechnological products.
• New communication channels and debates should be opened with participation of different sides such as consumers, researchers, authorities, industrial sectors, etc. to discuss impacts of this technology in human life, economy, and science.

This technology in the long term may provide innovative and economical development routes for human nutrition worldwide.

## Conclusion

Agriculture which is the only provider of human’s food that should produce from transitional and final inputs with well-known technologies. Thus, it is necessary to take a modern knowledge in agriculture. In spite of being relative advantages in agriculture process, still developing countries are suffering from lack of high importance of food products. Despite a lot of information about individual nanomaterials are available, but toxicity level of many NPs is still indefinable, thus the application of these materials is limited due to the lack of knowledge of risk assessments and effects on human health. Development of comprehensive database and alarm system, as well as international cooperation for regulation and legislation are necessary for exploitation of this technology.

## Author Contributions

RP, AB, and QN developed the idea and wrote the manuscript. All authors proofread and approved the final manuscript.

## Conflict of Interest Statement

The authors declare that the research was conducted in the absence of any commercial or financial relationships that could be construed as a potential conflict of interest.

## Footnotes

1 patentscope.wipo.int (accessed date: March 15, 2017).

2 hkc22.com
